# Painless Appendicitis: A Peculiar Surgical Case of Acute Granulomatous Appendicitis

**DOI:** 10.7759/cureus.95073

**Published:** 2025-10-21

**Authors:** Chrysanthi Karagianni, Ruby Barrick, Farida T Shamma, Panna K Patel

**Affiliations:** 1 Department of General and Colorectal Surgery, Furness General Hospital, Barrow-in-Furness, GBR

**Keywords:** acute appendicitis, atypical appendicitis, diagnosis of acute appendicitis, erythema nodosum, granulomatous appendicitis, granulomatous appendix, painless appendicitis, sarcoid-like reaction, sarcoidosis, sarcoidosis-like

## Abstract

Acute appendicitis is one of the most common surgical emergencies, typically presenting with right-sided abdominal pain, nausea, and anorexia. Painless appendicitis is very uncommon and poses a diagnostic challenge. This case report highlights an unusual presentation of painless granulomatous appendicitis. We present a female patient in her 20s with a history of erythema nodosum and systemic inflammatory symptoms who was incidentally diagnosed with acute retrocecal appendicitis on imaging. Despite radiological evidence, she denied abdominal pain or other classic symptoms. After a thorough rheumatologic evaluation, she underwent laparoscopic appendicectomy, which revealed a retrocecal, inflamed appendix. The histology report revealed granulomatous appendicitis, a condition likely linked to an underlying systemic inflammatory disorder such as sarcoidosis. Granulomatous appendicitis is a rare entity and, as it is associated with various conditions, further evaluation by the Rheumatology team is necessary. Painless appendicitis is uncommon but clinically important, and our case highlights the significance of maintaining a high suspicion index in atypical presentations to ensure timely diagnosis and management.

## Introduction

Acute appendicitis is one of the most common Emergency General Surgery pathologies worldwide, with approximately 50,000 appendicectomies performed annually in the United Kingdom alone [1]. Clinical diagnosis is often reliant on a thorough history and physical examination, supplemented by stratification scoring systems and imaging modalities. The classical clinical triad of migratory right iliac fossa (RIF) pain, nausea with vomiting, and anorexia is present in less than 50% of cases [2]. Despite the absence of classic clinical symptomatology, a degree of abdominal pain is almost always reported. The absence of pain (especially migratory RIF pain, rebound tenderness, and guarding) can be used as a negative diagnostic indicator of acute appendicitis [3].

A review of the literature reveals only a handful of published cases of painless acute appendicitis, representing probably less than 1% of published appendicitis case reports, with limited discussion regarding its management.

Granulomatous appendicitis, as seen in this case, is a rare histopathological subtype characterized by granuloma formation within the appendix, which may be idiopathic or secondary to systemic inflammatory or infectious causes. Recognition of this atypical entity is clinically important as it can mimic or coexist with other granulomatous diseases such as Crohn's disease, tuberculosis, or sarcoidosis [4].

This case report highlights a 20-year-old female patient with radiologically confirmed uncomplicated retrocecal appendicitis, who presented with atypical symptoms in the absence of RIF pain or tenderness.

## Case presentation

Initial presentation

**Figure 1:**
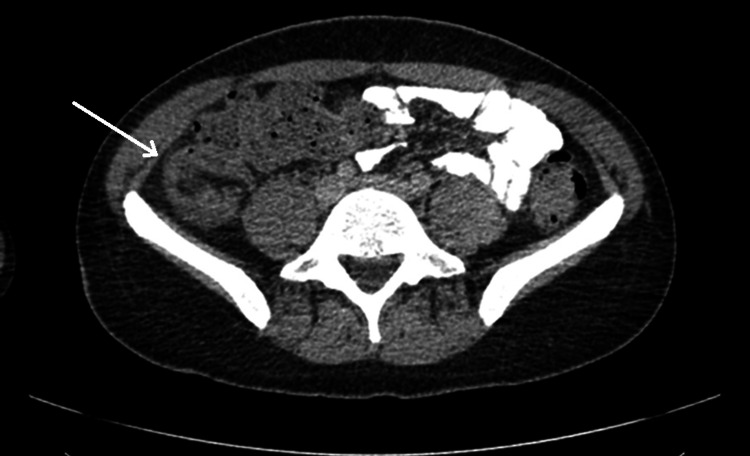
CT of chest, abdomen, and pelvis with contrast Axial image of CT chest, abdomen, and pelvis: the arrow points to a thickened, inflamed appendix in the right lower quadrant, measuring 12 mm in diameter, posterior to the cecum with periappendiceal fat stranding and small local lymph nodes, consistent with acute appendicitis. There is no evidence of collection, generalized peritonitis, free fluid, or free gas. Also, there were bilateral small-volume cervical lymph nodes that were nonspecific, bilateral tonsillar hypertrophy, but there was no evidence of lymphoma, lymphoproliferative disorder, or sarcoidosis (not seen in the figure) CT: computer tomography

A 20-year-old Caucasian female patient, with a background of previous recurrent urinary tract infections, fatigue, and recurrent erythema nodosum, presented to the Surgical Emergency Assessment Clinic with a three-month history of severe constipation requiring high-dose laxatives and enemas. The patient has no known allergies, has not undergone any previous surgeries, and is currently taking only a daily combined oral contraceptive pill. She had developed bilateral lower limb erythema nodosum on three separate occasions in the last six months prior to her presentation. The patient had an ongoing rheumatologic evaluation as an outpatient due to recurrent erythema nodosum, arthralgia, painless cervical lymphadenopathy, and intermittent fever with night sweats. Given her systemic inflammatory symptoms, she was being investigated for sarcoidosis. An outpatient computed tomography (CT) chest, abdomen, and pelvis that was performed the day before her presentation, as part of her Rheumatological workup, incidentally revealed uncomplicated acute appendicitis (Figure 1). The CT chest, abdomen, and pelvis revealed a thickened appendix, inflamed in the right lower quadrant (RLQ), measuring 12 mm in diameter, posterior to the cecum with periappendiceal stranding and small local lymph nodes, consistent with acute appendicitis. There was no evidence of collection, generalized peritonitis, free fluid, or free gas. The terminal ileum and cecal pole were normal, and there were no features of inflammatory disease. Despite radiologic confirmation of appendicitis, she denied any right-sided abdominal pain, nausea, vomiting, or anorexia. She was seen two months prior by the surgical team with severe constipation and fecal impaction requiring treatment with Picolax® (bowel preparation with sodium picosulfate, magnesium oxide, light, and citric acid). During that time, she had a flexible sigmoidoscopy that did not reveal any abnormalities. From her detailed background history, she has a strong family history of autoimmune disease, with her mother being diagnosed with myasthenia gravis and her sister with alopecia areata.

Examination and admission

**Figure 2:**
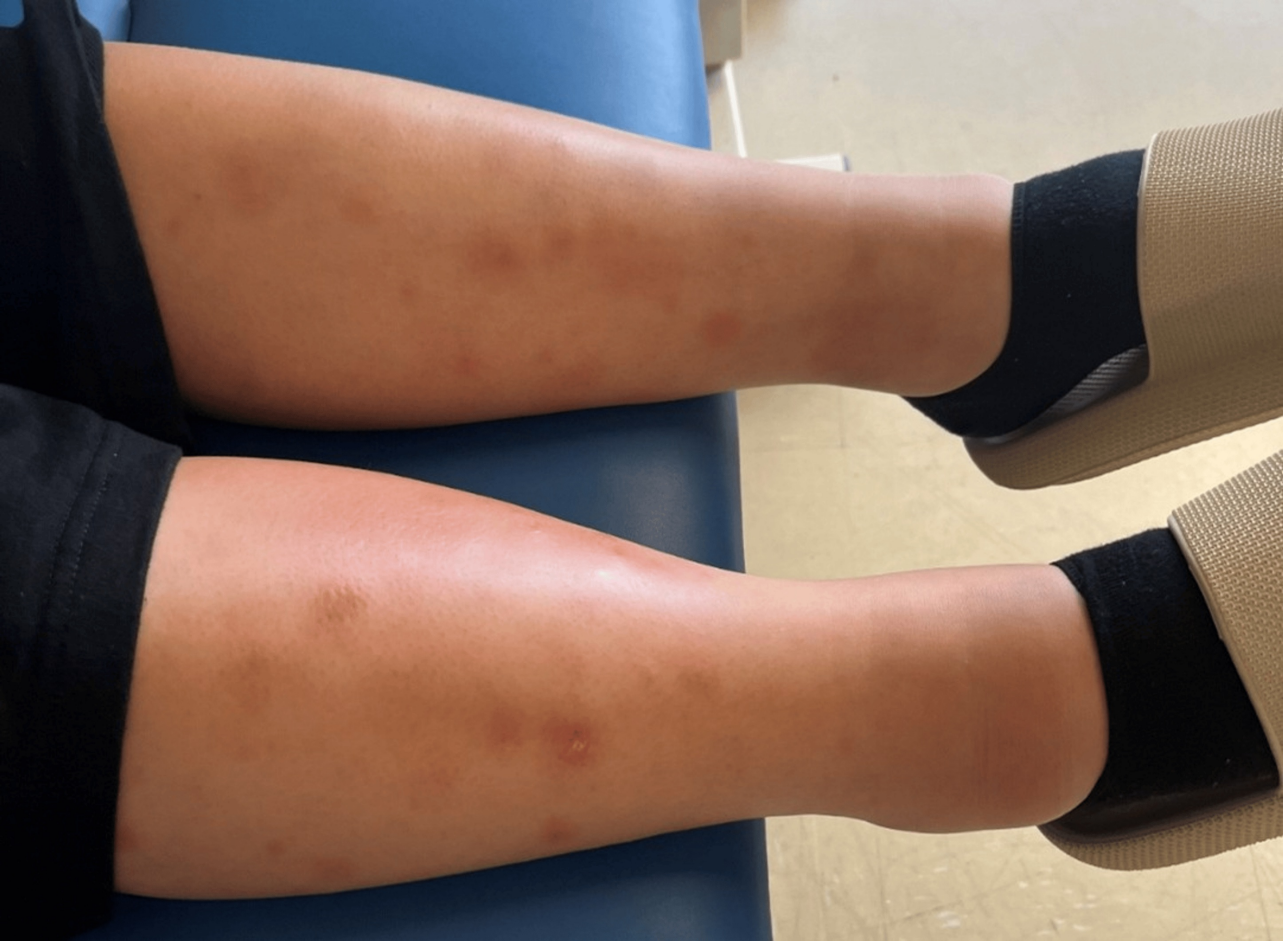
Bilateral erythema nodosum Image courtesy: Written, informed consent to publish this article has been obtained from the patient

On admission, her vital signs were stable, with a low-grade fever (37.4°C). Her head-to-toe evaluation revealed enlarged tonsils without exudate, small nontender cervical lymph node enlargement, and arthralgia. Her chest auscultation was clear without any added sounds, and her cardiovascular system was intact without any abnormalities. Her abdominal examination revealed a soft, nontender, nondistended abdomen with audible bowel sounds. Notably, bilateral lower limb swelling with tender erythematous nodules was present with mild pitting edema (Figure 2). She did not report any joint swelling, recurring mouth ulcers, or symptoms related to the eyes, mouth, or urinary tract, or mouth dryness at the moment. There were no other rashes or other peripheral lymphadenopathy.

The initial investigation plan was formulated jointly with the Rheumatology Team in view of the patient’s multisystemic presentation, which included arthralgia, fever, cervical lymphadenopathy, erythema nodosum, and, subsequently, painless CT-confirmed appendicitis. The working differential diagnosis encompassed infectious, autoimmune, and inflammatory conditions, including sarcoidosis, systemic lupus erythematosus, and vasculitis, as well as potential thromboembolic disease. Consequently, a comprehensive panel of investigations was undertaken, including full blood count, renal, liver, and bone profiles, inflammatory markers (C-reactive protein, erythrocyte sedimentation rate, and procalcitonin), thyroid function, immunoglobulins, D-dimer, and urinalysis with microscopy and culture to exclude infection. Bilateral lower limb Doppler ultrasonography was performed to rule out deep vein thrombosis, given the association between systemic inflammation and thromboembolic events. Procalcitonin was included to aid in differentiating bacterial infection from noninfectious inflammatory processes, supporting the broader diagnostic evaluation in the absence of overt sepsis. Blood cultures for aerobic and anaerobic bacteria were taken when the patient was febrile (Table 1).

**Table 1:** Preoperative investigations US: ultrasound; DVT: deep vein thrombosis

Investigation test	Result	Units	Reference range
White cell count	9	× 10^9^/L	4.0-10.0
Neutrophils	6	× 10^9^/L	2.0-7.5
C-reactive protein	77.5	mg/L	<10
Erythrocyte sedimentation rate	39	mm/hour	0-36.0
Procalcitonin	<0.5	ng/mL	<0.5
Fecal calprotectin	329	μg/g	0-50.0
IgG	15.37	g/L	5.4-16.1
IgM	1.7	g/L	0.5-2.0
IgA	4.52	g/L	0.7-2.5
Urine dip-stick	Negative		
Midstream urine culture	No growth (negative)		
Blood cultures (aerobic and anaerobic)	No growth after five days (negative)		
Doppler US scan bilateral legs	Negative for DVT		

After her assessment by the Surgical Team, given her low-grade fever, she was prescribed intravenous (IV) antibiotics (amoxicillin 1 g three times a day, metronidazole 500 mg three times a day, and gentamicin 5 mg/kg every 24 hours) and IV fluid replacement.

Admission and decision to operate

Given the discordance between imaging findings and clinical presentation, an urgent rheumatology review was sought. Subsequent abdominal ultrasound (US) reaffirmed the presence of acute retrocecal appendicitis. Given the presence of systemic inflammatory symptoms (fever, night sweats) and the presence of appendicitis in two imaging modalities (CT and US scans), surgical intervention was advised.

**Figure 3:**
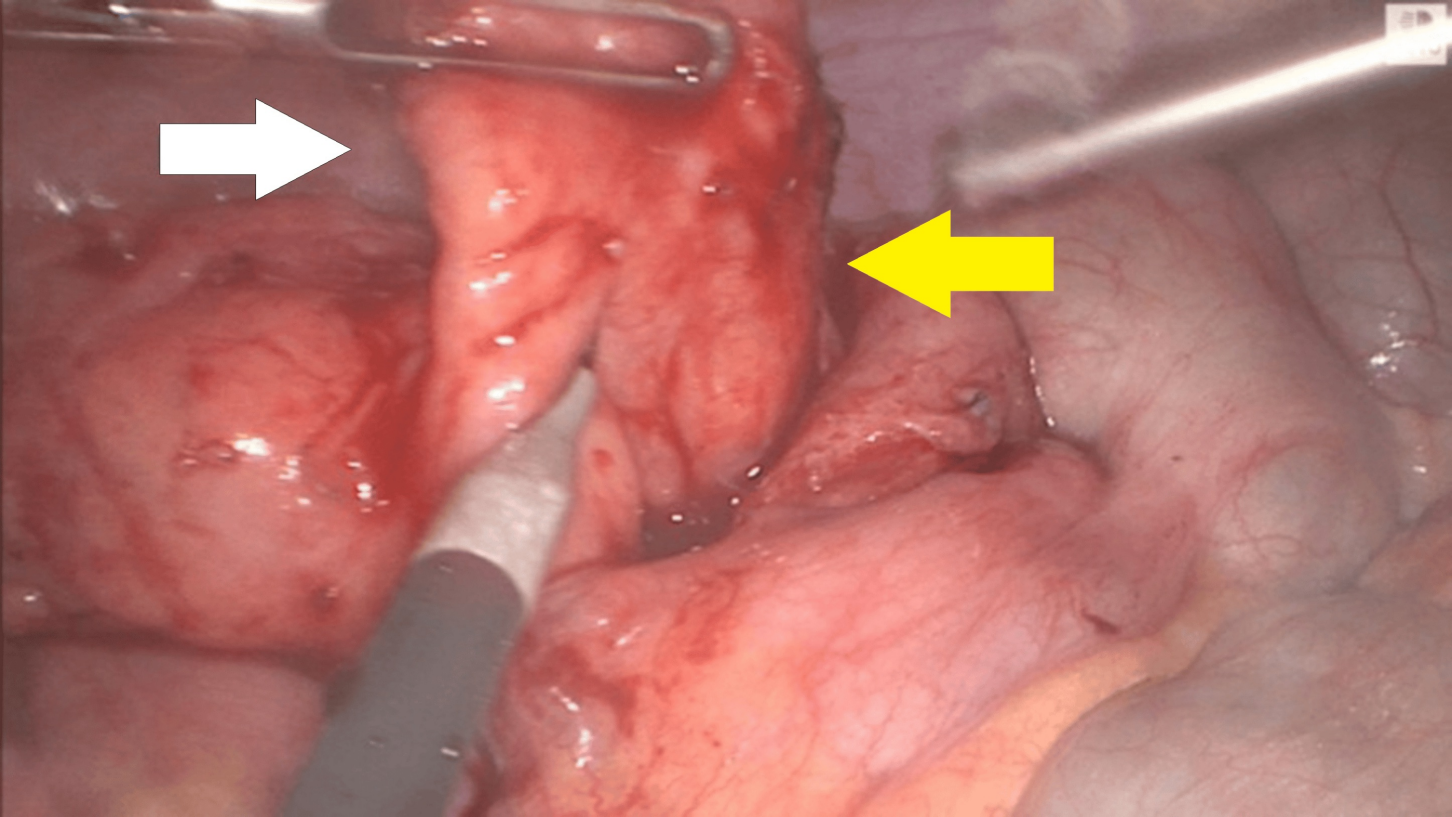
The inflamed appendix (yellow arrow) and the thickened mesoappendix (white arrow) (intraoperative picture obtained from laparoscopic stack system)

Following an extensive discussion regarding risks and benefits, the patient consented to a laparoscopic appendicectomy. Intraoperatively, the appendix was found to be markedly thickened, inflamed, retrocecal, and densely adherent to the lateral abdominal wall (Figure 3). Intraoperatively, the terminal ileum and distal cecum were also densely adhered to the lateral abdominal wall. The appendix was long with a long mesoappendix. It was dissected with difficulty from the lateral abdominal wall. The base was assessed, defined, and resected with an articulating endoscopic/laparoscopic stapler with purple load (EndoGIA^TM^ 45 mm, Medtronic, Minneapolis, Minnesota). The mesoappendix was divided with an electrothermal bipolar vessel sealing device (LigaSure^TM^, Medtronic) to control hemorrhage from the appendiceal artery. The appendiceal stump was not buried, and after copious lavage with saline, an 18 Fr Robinson surgical drain was left in the pelvis. The histopathological macroscopic analysis demonstrated an appendix measuring 95 mm in length and 12 mm in luminal diameter, with an attached mesoappendix measuring 45 mm to a depth of 25 mm. The lumen was dilated and contained hemorrhagic material. The wall was thickened. Microscopically, the histology report indicated an appendix with luminal hemorrhage and erosion of the surface epithelium. There was mixed inflammation comprising lymphocytes, a few neutrophils, and eosinophils extending through the wall with a fair number of lymphoid aggregates. There were a few discrete appearing granulomas without any definitive central necrosis. They were seen in the mucosa, some in the lymphoid follicles, and in the submucosa. There were no foreign bodies seen, and there was no dysplasia or evidence of malignancy. Special stains (Ziehl-Neilson, periodic acid Schiff, and Grocott) did not demonstrate any infectious agents. The conclusion was granulomatous appendicitis, and the differential diagnosis was wide and included infections (special stains for acid-fast bacteria and fungi are negative), foreign bodies (none demonstrated), diverticula with inflammation (none demonstrated), Crohn’s disease (requiring clinical and radiological correlation), and, lastly, after exclusion, idiopathic granulomatous appendicitis.

Postoperative course

The patient recovered uneventfully from the laparoscopic appendicectomy and was discharged on postoperative day 4 with a course of oral antibiotics. The erythema nodosum and bilateral leg edema resolved rapidly after the operation. For the remainder of her hospital stay, she remained afebrile and without arthralgia or other sarcoid-like reaction-related symptoms. Her night sweats and fatigue completely subsided after the operation.

Rheumatological follow-up

At her outpatient rheumatology follow-up, two months after her laparoscopic appendicectomy, her full rheumatological presentation was summarized. Her presentation included previous recurrent erythema nodosum, ankle swelling and pain, peripheral arthralgias, recurrent urinary infections, constipation, left eye blurring, mouth ulcers, cervical lymphadenopathy, and granulomatous appendicitis. Since her operation, her night sweats, fever, and fatigue had resolved. She denied any weight loss, hair loss, mucosa dryness, Raynaud symptoms, or genital ulceration. On further evaluation of her family history, she could not identify any family members of Southern European, Mediterranean, or Middle Eastern descent. She was thoroughly examined and was found to be well perfused with no evidence of synovitis or rashes. She did not have erythema nodosum, but there was hyperpigmentation on the skin of her lower legs. She had small, nontender cervical lymphadenopathy. Her chest was clear, and her abdominal examination was unremarkable. She had further outpatient investigations, including an ultrasound of her neck to reassess her lymphadenopathy, a repeat fecal calprotectin test, and a full colonoscopy. The original differential diagnosis was sarcoidosis or sarcoid-like syndrome, but it was important to rule out inflammatory bowel disease (Crohn's disease of the appendix) or Behcet's disease (a chronic inflammatory condition leading to painful mouth and genital ulcers, uveitis, and arthritis). Her neck ultrasound did not reveal any evidence of Sjogren's syndrome or sarcoid-like changes, and findings were in keeping with reactive lymph nodes. A full colonoscopy was achieved, albeit challenging due to a loopy bowel, and the findings were completely normal. Her fecal calprotectin remained within normal limits. She was seen subsequently in clinic, after the results of the requested tests, and given the lack of further symptoms, she was deemed to have a self-limiting granulomatous disorder, likely sarcoidosis. Given the absence of current disease flare-ups, she remains on a patient-initiated follow-up plan with Rheumatology.

## Discussion

It is a global consensus that, despite advances in diagnostic imaging and the use of biochemical markers, which may support the differential diagnosis, acute appendicitis is still considered to be a clinical diagnosis with abdominal pain as its cardinal symptom [5].

In 1889, McBurney, a surgeon and professor at the American College of Surgeons and Physicians, identified an anatomical landmark associated with the pain indicative of appendicitis. He described this point as being located one-third of the distance from the anterior superior iliac spine to the linea alba at the level of the umbilicus. This landmark was later named McBurney’s point, and tenderness in that point has been the most indicative symptom for diagnosing appendicitis [6].

Acute appendicitis is considered one of the most common causes of acute abdomen in children and adults. It typically manifests as vague or diffused abdominal pain, which can later migrate to the RLQ of the abdomen due to irritation of the peritoneum. The classical clinical presentation includes the migratory RIF pain, often associated with nausea, vomiting, or anorexia [7]. Atypical presentations can pose a clinical challenge, leading to delays in diagnosis and treatment.

Unusual presentations of appendicitis are well-documented, particularly in immunocompromised patients and those with retrocecal appendices. Nonetheless, truly painless presentations, like in our patient, are exceedingly rare, with limited reports in the literature. In most atypical cases, patients still experience gastrointestinal symptoms, such as nausea, vomiting, or anorexia, even if abdominal pain is minimal or absent [2,8].

Right-sided abdominal pain remains the predominant feature of appendicitis, though the exact prevalence varies among different studies. In a systematic review and meta-analysis that evaluated the diagnostic features of appendicitis according to the Alvarado score, it reported that approximately 83% of patients with confirmed appendicitis presented with RLQ pain at admission [9]. These findings align with data from another individual single-center surgical series where RLQ pain and tenderness were observed in up to 98% of the cases [10].

Histopathological examination in this case demonstrated granulomatous appendicitis, an uncommon entity reported in only 0.1%-2% of appendicectomy specimens. In one large histopathological study analyzing more than 24,000 appendicectomy samples, the prevalence of granulomatous appendicitis was approximately 0.19% [11]. The differential diagnosis for this finding is extensive. It may be idiopathic or part of a systemic disease such as Crohn's disease, sarcoidosis, or idiopathic granulomatous appendicitis. More commonly, it is secondary to infectious etiologies, including bacterial (e.g., *Mycobacterium tuberculosis*, *Yersinia enterocolitica*, Actinomyces, Brucella, and Campylobacter), fungal (e.g., Histoplasma, Blastomyces, Candida), or parasitic infections (e.g., *Enterobius vermicularis*, Schistosoma) [12-14]. A comprehensive clinical assessment and correlation are essential to avoid delays, misdiagnosis, and inappropriate therapeutic plans.

In our case, it was considered that the granulomatous appendicitis was associated with a systemic sarcoid-like syndrome, which is thought to be a granulomatous disease of unknown etiology [15]. While it predominantly presents with pulmonary and lymphatic involvement, intestinal manifestations are evident in rare cases, accounting for less than 1% of sarcoidosis presentations, and appendiceal involvement is particularly rare [16]. The exceptional rarity of this case, both as a painless presentation of appendicitis and histological evidence of granulomatous appendicitis secondary to sarcoid-like syndrome, emphasizes its clinical importance.

From a clinical perspective, this case highlights two main points. First, the absence of pain should not entirely exclude appendicitis in the appropriate clinical context; clinicians must remain vigilant when evaluating patients with unexplained gastrointestinal symptoms, even if classical features are absent. Second, routine histopathological analysis of appendicectomy specimens is still essential, as it may reveal unexpected underlying systemic conditions. In this patient, the finding of granulomatous appendicitis prompted further investigation and ultimately led to the diagnosis of intestinal sarcoidosis.

The main limitation of this case report is that it presents a single case in our unit; therefore, causality cannot be inferred. However, it illustrates a common diagnostic challenge with real implications for clinical practice.

## Conclusions

This case highlights a rare and diagnostically challenging presentation of painless appendicitis with histologically confirmed granulomatous inflammation. The absence of pain in a traditionally painful condition underscores the importance of maintaining a high index of suspicion, especially in patients with atypical systemic symptoms.

The case also raises awareness of granulomatous appendicitis as a potential manifestation of systemic inflammatory disorders such as sarcoidosis. Given the rarity of this entity, further studies are warranted to elucidate the pathophysiological mechanisms linking granulomatous appendicitis to systemic disease. Clinicians should remain vigilant for unusual presentations and consider appendicitis even in the absence of classical symptoms, particularly when radiologic findings suggest inflammation.
